# Enhancing Root Canal Disinfection with Er:YAG Laser: A Systematic Review

**DOI:** 10.3390/dj13030101

**Published:** 2025-02-26

**Authors:** Jakub Fiegler-Rudol, Zuzanna Grzech-Leśniak, Marcin Tkaczyk, Kinga Grzech-Leśniak, Anna Zawilska, Rafał Wiench

**Affiliations:** 1 Department of Periodontal and Oral Mucosa Diseases, Faculty of Medical Sciences in Zabrze, Medical University of Silesia, 40-055 Katowice, Poland; mtkaczyk@sum.edu.pl (M.T.); rwiench@sum.edu.pl (R.W.); 2 Faculty of Dentistry, Wroclaw Medical University, 50-425 Wroclaw, Poland; zuzanna.grzech-lesniak@student.umw.edu.pl; 3 Dental Surgery Department, Wroclaw Medical University, 50-425 Wroclaw, Poland; 4 Department of Periodontics, School of Dentistry, Virginia Commonwealth University, Richmond, VA 23284, USA; 5 Department of Conservative Dentistry and Endodontics, Faculty of Medical Sciences in Zabrze, Medical University of Silesia, 40-055 Katowice, Poland; azawilska@sum.edu.pl

**Keywords:** Er:YAG laser, root canal disinfection, photoacoustic streaming, PIPS, SWEEPS, smear layer removal, endodontics

## Abstract

**Background:** The quest for minimally invasive disinfection in endodontics has led to using Erbium:Yttrium-Aluminum-Garnet (Er:YAG) lasers. Conventional approaches may leave bacterial reservoirs in complex canal anatomies. Er:YAG’s strong water absorption generates photoacoustic streaming, improving smear layer removal with lower thermal risk than other laser systems. **Methods:** This systematic review followed PRISMA 2020 guidelines. Database searches (PubMed/MEDLINE, Embase, Scopus, Cochrane Library) identified studies (2015–2025) on Er:YAG laser-assisted root canal disinfection. Fifteen articles met the inclusion criteria: antibacterial efficacy, biofilm disruption, or smear layer removal. Data on laser settings, irrigants, and outcomes were extracted. The risk of bias was assessed using a ten-item checklist, based on guidelines from the Cochrane Handbook for Systematic Reviews of Interventions. **Results:** All studies found Er:YAG laser activation significantly improved root canal disinfection over conventional or ultrasonic methods. Photon-induced photoacoustic streaming (PIPS) and shock wave–enhanced emission photoacoustic streaming (SWEEPS) yielded superior bacterial reduction, especially apically, and enabled lower sodium hypochlorite concentrations without sacrificing efficacy. Some research indicated reduced post-operative discomfort. However, protocols, laser parameters, and outcome measures varied, limiting direct comparisons and emphasizing the need for more standardized, long-term clinical trials. **Conclusions:** Er:YAG laser-assisted irrigation appears highly effective in biofilm disruption and smear layer removal, supporting deeper irrigant penetration. While findings are promising, further standardized research is needed to solidify guidelines and confirm Er:YAG lasers’ long-term clinical benefits.

## 1. Introduction

### 1.1. Rationale

Root canal therapy aims to eradicate microbial infection and prevent reinfection within the intricate canal system [1,2]. Despite advances in endodontic techniques, effective disinfection remains a critical challenge [1,2]. Conventional approaches—such as mechanical instrumentation coupled with chemical irrigants like sodium hypochlorite (NaOCl) and ethylenediaminetetraacetic acid (EDTA)—often leave behind residual bacteria, debris, and smear layers in complex canal anatomies [3]. These shortcomings can lead to persistent infections, post-operative discomfort, and, ultimately, treatment failure if not adequately addressed [4]. Consequently, the pursuit of more efficient and minimally invasive disinfection methods has garnered significant interest within the endodontic community [5,6]. Eradicating bacteria and removing debris from the intricately shaped root canal system are paramount to ensuring successful endodontic outcomes [7]. Conventional mechanical instrumentation, while essential, often fails to thoroughly clean recesses such as isthmuses, lateral canals, and deep dentinal tubules, leaving residual bacterial colonies that can compromise healing and lead to long-term treatment failures [8]. Traditional chemical irrigants, including sodium hypochlorite (NaOCl) and ethylenediaminetetraacetic acid (EDTA), play a significant role in dissolving organic tissue and removing smear layers, but their effectiveness is limited by factors such as contact time, canal anatomy, and the capability of the irrigating fluid to penetrate microchannels [9,10,11]. Sodium hypochlorite is toxic, which necessitates careful handling to avoid potential tissue damage and adverse effects [9,10,11]. In pursuit of more efficient and predictable disinfection, laser-based methods have garnered increasing attention [11,12]. Among them, the Erbium: Yttrium-Aluminum-Garnet (Er:YAG) laser distinguishes itself by operating at a wavelength (2940 nm) that is highly absorbed by water molecules [13]. This characteristic promotes the generation of strong photoacoustic streaming and cavitation effects when the laser interacts with irrigating solutions inside the root canal system, creating shockwaves powerful enough to penetrate areas not easily reached by standard methods [13,14,15]. Consequently, Er:YAG laser-activated irrigation may bolster bacterial reduction, augment smear layer removal, and improve dentinal tubule penetration [16]. Moreover, Er:YAG lasers typically produce less heat compared to other laser systems (e.g., diode, Nd:YAG) due to their shorter depth of penetration and high water absorption [17]. This reduced thermal effect on the dentin surface lowers the risk of structural damage or compromised mechanical properties, making Er:YAG lasers especially suitable for delicate or thin-walled roots. Initial studies also suggest potential advantages in pediatric endodontics, where preserving tooth structure is vital, and in regenerative or revascularization procedures aimed at stimulating tissue healing within the root canal space [18,19,20,21,22,23,24]. However, despite these promising attributes, considerable heterogeneity exists in reported protocols, laser parameters, study designs, and measured outcomes, underscoring the need for a systematic review to establish clearer insights into the clinical utility and optimal application strategies of Er:YAG laser technology in root canal disinfection [12,13,14,15,16,17,18,19,20,21,22,23,24,25,26,27,28]. Laser-Activated Irrigation (LAI) using Erbium:Yttrium-Aluminum-Garnet (Er:YAG) lasers has emerged as a promising technique for enhancing root canal disinfection through Photon-Induced Photoacoustic Streaming (PIPS) and Shock Wave-Enhanced Emission Photoacoustic Streaming (SWEEPS), both of which improve smear layer removal and biofilm disruption [16,17,18,19,20].

### 1.2. Objectives

This systematic review aims to consolidate and evaluate the evidence on Er: YAG laser-assisted root canal disinfection by comparing its efficacy in bacterial reduction, biofilm eradication, and smear layer removal to both conventional and other laser-based methods. The study seeks to address the following key research question: Does Er:YAG laser technology provide superior root canal disinfection compared to conventional disinfection methods, and how can it be optimised for clinical application? Moreover, by identifying methodological inconsistencies and gaps in current research, this review provides recommendations for optimizing clinical procedures and refining experimental designs. Finally, it examines whether Er:YAG technology can be feasibly integrated into standard practice.

### 1.3. Justification

The need for conducting this study is underscored by the persistent challenges in achieving optimal root canal disinfection, a crucial factor in the long-term success of endodontic treatments. Conventional irrigation techniques often fail to fully eliminate bacterial biofilms and debris from complex root canal anatomies, leading to reinfection and treatment failures. The emergence of Er:YAG laser technology presents a promising alternative, offering superior antibacterial effects, enhanced smear layer removal, and improved penetration of irrigants into dentinal tubules. This topic is highly relevant to the field of odontology, particularly in endodontics and periodontics, where bacterial control is paramount. In endodontics, the ability of Er:YAG lasers to enhance disinfection could significantly improve clinical outcomes, reducing the likelihood of post-treatment infections and enhancing the longevity of root canal treatments. In periodontics, Er:YAG lasers have demonstrated potential in minimally invasive periodontal therapy, promoting better healing outcomes by effectively targeting bacterial deposits without causing excessive thermal damage. Given the growing interest in laser-assisted endodontic procedures and the need for evidence-based guidelines, this systematic review will contribute to the field by consolidating existing research, identifying gaps in knowledge, and providing insights into the optimal application of Er:YAG laser technology in clinical practice. The findings of this study could inform future clinical protocols and encourage broader adoption of laser-assisted irrigation techniques, ultimately improving patient outcomes in both endodontic and periodontal treatments.

## 2. Methods

### 2.1. Focused Question

A systematic review was conducted following the PICO framework [29], structured as follows: Among patients undergoing root canal therapy (Population), does the use of Er: YAG laser technology (Intervention) result in superior disinfection outcomes compared to conventional disinfection methods (Comparison) in improving the effectiveness of root canal disinfection (Outcome)?

### 2.2. Search Strategy

This systematic review, registered with PROSPERO under the number CRD42025638319 [30], adhered to the PRISMA 2020 guidelines to ensure thorough and transparent reporting [31]. A detailed search strategy was employed across multiple databases, including PubMed/Medline, Embase, Scopus, and the Cochrane Library, using a set of predefined keywords [28]. Independent searches were conducted by three researchers, following uniform protocols. The review included English-language studies published between 1 January 2015, and 3 January 2025, based on specific eligibility criteria. Two reviewers independently screened articles at the title, abstract, and full-text levels. Additionally, reference lists of selected studies were reviewed to uncover further relevant publications. The search syntax used to obtain these studies is available in Table 1.

### 2.3. Selection of Studies

During the selection phase of this systematic review, the four reviewers independently evaluated the titles and abstracts of the retrieved studies to maintain impartiality in the inclusion process. Any disagreements regarding a study’s eligibility were resolved through collaborative discussion until a mutual agreement was reached. This meticulous approach, aligned with PRISMA guidelines, ensured the inclusion of only the most relevant and methodologically robust studies, enhancing the reliability and reproducibility of the review [31].

The inclusion criteria for this systematic review focused on studies investigating the use of Er:YAG laser technology in root canal disinfection, encompassing both in vitro experiments and animal research. Studies that explored the combination of Er:YAG laser treatment with other antimicrobial or adjunctive therapies were also eligible for inclusion. Research with control groups comparing Er:YAG laser disinfection to standard mechanical cleaning, alternative non-surgical techniques, or no treatment was included, as well as studies directly comparing the effectiveness of Er:YAG lasers with other non-surgical approaches. Long-term studies evaluating the sustained impact of Er:YAG laser technology on key outcomes, such as bacterial reduction, infection control, and healing of periapical tissues, were prioritized. Only publications meeting predefined quality standards (Table 2) and specifically addressing the effectiveness or role of Er:YAG lasers in improving root canal disinfection were considered for inclusion.

Exclusion criteria for this systematic review encompassed grey and unpublished literature, such as dissertations, conference abstracts, theses, and non-peer-reviewed materials, as well as articles published in languages other than English. Duplicate publications or those with identical ethical approval numbers were excluded. Studies that focused on topics outside the scope of root canal disinfection with Er:YAG laser technology were not included, along with those using alternative laser systems or methods unrelated to Er:YAG. In vitro studies that did not simulate clinically relevant dental conditions or address pertinent microbial strains were excluded. Additionally, non-primary data formats, such as case reports, case series, narrative reviews, systematic reviews, editorials, and books, were omitted, as were studies lacking a control or comparison group. Finally, studies involving non-therapeutic applications of Er:YAG lasers were not considered.

### 2.4. Risk of Bias in Individual Studies and Quality Assessment

To initiate the study selection process for this systematic review, each reviewer conducted an independent evaluation of the titles and abstracts of articles related to Er:YAG applications in root canal treatment, aiming to minimize bias. Consistency in decision-making was ensured by calculating inter-rater reliability using Cohen’s kappa statistic [32]. Any disagreements regarding a study’s inclusion were addressed through in-depth discussions, ultimately resulting in a unanimous resolution.

The methodological quality of the included studies was independently assessed by two reviewers to ensure reliability and objectivity. The evaluation focused on key aspects of Er:YAG laser disinfection, assigning a score of 1 for “yes” and 0 for “no” responses to the following criteria: (1) Was the study design clearly defined and appropriate for assessing Er:YAG laser disinfection? (2) Were the laser parameters (e.g., wavelength, power output, energy settings, pulse duration, and repetition rate) explicitly stated and justified? (3) Did the study include both positive and negative control groups for comparison? (4) Were root canal samples standardized in size, anatomy, and preparation protocols? (5) Was the laser application protocol clearly described, including duration, delivery method, and number of applications? (6) Were primary and secondary outcome measures (e.g., bacterial reduction, disinfection efficiency) clearly defined and appropriate? (7) Was there complete reporting of outcome data without missing or inconsistent information? (8) Were numerical results accompanied by appropriate statistical analyses? (9) Was the study conducted independently of its funding sources to avoid conflicts of interest? (10) Were participants, investigators, or evaluators blinded to treatment allocations to minimize bias? Based on the total “yes” responses, each study’s risk of bias was categorized as high (0–3 points), moderate (4–6 points), or low (7–10 points), following the Cochrane Handbook for Systematic Reviews of Interventions [33]. This comprehensive quality assessment ensured that only studies with robust and reliable methodologies were included in the review, enhancing the validity and credibility of its findings.

Table 2 presents a comprehensive assessment of bias risk across the ten studies included in the final analysis. Studies were required to achieve a minimum score of six points based on the predefined evaluation criteria to qualify for inclusion. All studies met the criteria for low bias risk. Notably, no studies fell into the categories of moderate or high bias risk, underscoring the dependability of the systematic review’s conclusions.

**Table 2:** The outcomes of the quality evaluation and bias risk assessment for the studies.

Author and Year	1	2	3	4	5	6	7	8	9	10	Total	Risk
Afkhami et al. 2021 [34]	1	1	1	1	1	1	1	1	1	1	10	Low
Bao et al. 2024 [35]	1	1	0	1	1	1	1	1	0	0	7	Low
Deleu et al. 2015 [36]	1	1	1	1	0	1	1	1	1	1	9	Low
Ozbay et al. 2018 [37]	1	1	1	1	1	1	1	1	1	1	10	Low
Lei et al. 2022 [38]	1	1	1	1	1	1	1	1	0	1	9	Low
Liu et al. 2023 [39]	1	1	0	1	1	1	0	1	1	0	7	Low
Mandras et al. 2020 [40]	1	1	1	1	1	1	1	1	1	1	10	Low
Murugesh Yavagal et al. 2021 [41]	1	1	0	1	1	1	0	1	1	0	7	Low
Neelakantan et al. 2015 [42]	1	1	1	1	1	1	1	1	1	0	9	Low
Rahmati et al. 2022 [43]	1	1	1	1	1	1	1	1	1	1	10	Low
Shan et al. 2022 [44]	1	1	1	1	1	1	1	1	1	0	9	Low
Todea et al. 2018 [45]	1	1	0	1	1	1	1	1	0	1	8	Low
Yang et al. 2024 [46]	1	1	1	1	1	1	1	1	1	1	10	Low
Zhao et al. 2024 [47]	1	1	1	1	1	1	1	1	1	1	10	Low

### 2.5. Data Extraction

After finalizing the selection of relevant studies the two reviewers systematically extracted detailed information from each included article. This process involved recording bibliographic details such as the lead author’s name and the year of publication, the study design, the specific dental conditions addressed, and the types of experimental and control groups employed. They also documented the duration of follow-up periods, measured outcomes related to root canal disinfection effectiveness, and technical specifications of the Er:YAG laser systems used, including wavelength, power settings, pulse duration, and delivery methods. Additionally, the reviewers noted any adjunctive treatments or methodologies incorporated into the studies, such as irrigation solutions or additional antimicrobial protocols. This meticulous and comprehensive data extraction ensured that all relevant variables were thoroughly captured, allowing for a detailed analysis of the efficacy and operational parameters of Er:YAG laser technology in root canal disinfection. This rigorous approach facilitated a deeper understanding of the clinical applications of Er:YAG lasers, thereby enhancing the reliability and scope of the systematic review’s findings.

## 3. Results

### 3.1. Study Selection

**Figure 1:**
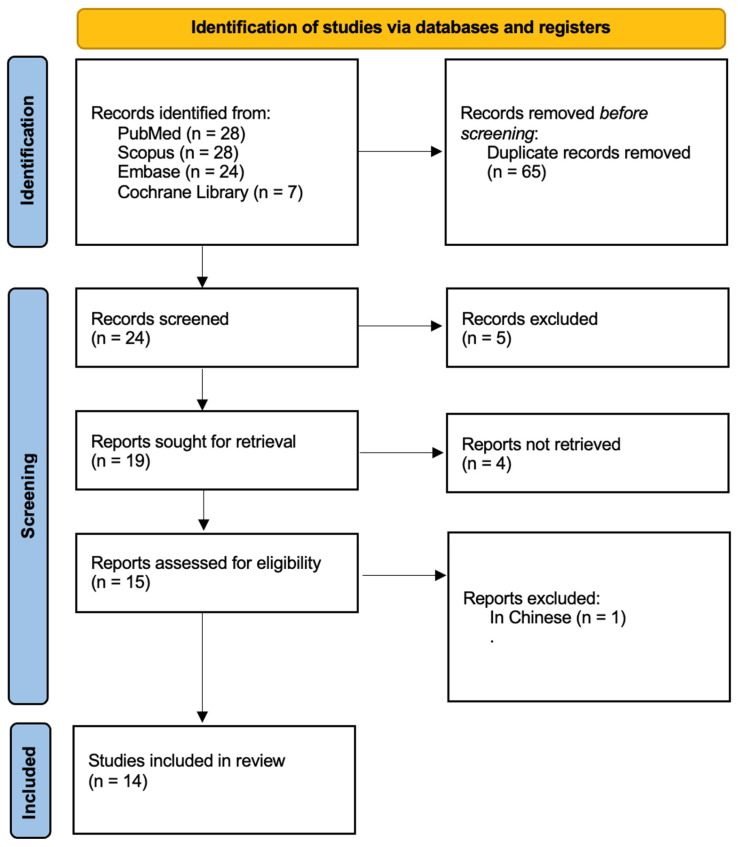
Prisma 2020 flow diagram.

Figure 1 outlines the research workflow, carefully structured in accordance with PRISMA guidelines [31]. The process commenced with an initial literature search, yielding 87 articles. After removing duplicate entries, 24 records were retained. A rigorous evaluation of titles and abstracts narrowed the selection to 15 studies, all of which underwent full-text review. One article was excluded as it did not have an English version. Ultimately, 14 studies published were included in the final analysis. Detailed characteristics of these studies are summarized in Table 3.

### 3.2. Data Presentation

Data from the 15 studies that met the inclusion criteria were meticulously extracted and systematically organised into Table 3, Table 4 and Table 5. These tables offer a comprehensive summary of each study, highlighting critical details such as the specifications of the light sources utilised and the properties of riboflavin as a photosensitiser in PDT protocols. This structured format facilitates in-depth comparisons and detailed analyses of Er:YAG laser applications across the included studies, providing a strong foundation for evaluating its effectiveness and methodological approaches in managing dental conditions.

### 3.3. General Characteristics of the Included Studies

Table 3, Table 4 and Table 5 present an overview of the key characteristics of the 15 studies.

### 3.4. Main Study Outcomes

Er:YAG laser technology—particularly photon-induced photoacoustic streaming (PIPS) and shock wave-enhanced emission photoacoustic streaming (SWEEPS)—has consistently demonstrated superior root canal disinfection compared to conventional methods, achieving up to 91.03% bacterial reduction and effectively removing biofilms from complex canal regions [34,35]. Studies revealed enhanced debris and smear layer removal [36], with Er:YAG laser activation outperforming Nd:YAG in facilitating sealer penetration [37]. Even at lower concentrations, sodium hypochlorite-maintained disinfection efficacy when activated by SWEEPS [38], and LAI-PIPS surpassed ultrasonic and syringe irrigation by producing stronger shockwave-like effects [39]. PIPS also demonstrated improved antibacterial outcomes with less post-operative discomfort [40] and complete elimination of Enterococcus faecalis in primary teeth [41]. When combined with specific irrigant protocols, the Er:YAG laser effectively disrupted mature biofilms [42], removed the smear layer while sealing dentinal tubules, and enhanced stem cell adhesion, unlike MTAD treatment [43]. Its efficiency remained high regardless of minimally invasive or conventional canal access [44], as it facilitated thorough cleaning across all canal sections without structural damage [45]. Furthermore, pairing the Er:YAG laser with photodynamic therapy led to 99.97% bacterial reduction [46], and PIPS using low-concentration NaOCl surpassed conventional needle irrigation in eliminating bacteria and improving clinical outcomes [47]. Table 4 presents the main outcomes of the included studies. Table 5 shows the parameters of the light sources used in this study.

## 4. Discussion

### 4.1. Results in the Context of Other Evidence

Er:YAG laser activation shows remarkable synergy with various irrigants, including silver nanoparticles and sodium hypochlorite, producing powerful photoacoustic shockwaves that increase bacterial reduction in complex canal anatomies [34,45]. It consistently outperforms or closely rivals conventional needle irrigation, passive ultrasonic irrigation, and sonic activation by achieving deeper irrigant penetration and superior biofilm removal, especially in the apical third [35,47]. The generation and implosion of vapor bubbles (cavitation) at the Er:YAG laser tip creates strong fluid dynamics that effectively flush debris from irregular canal walls, surpassing the cleaning capacity of manual-dynamic methods and diode lasers [35]. Activation with Er:YAG lasers also proves more efficient in removing the smear layer and enhancing sealer penetration compared to Nd:YAG laser treatments, particularly in curved root canals [37,42]. By employing Shock Wave Enhanced Emission Photoacoustic Streaming (SWEEPS), practitioners can use lower NaOCl concentrations without sacrificing antimicrobial efficacy, thus reducing the risk of tissue damage [38]. Photon-induced photoacoustic streaming (PIPS), especially when operating at subablative parameters, excels at cleaning beyond fractured instruments and in narrow or complex canal configurations by creating more vigorous bubble collapse and shockwaves [39,45]. In pediatric endodontics, PIPS has demonstrated near-complete bacterial elimination against Enterococcus faecalis in primary teeth, underscoring its potential for minimally invasive yet highly effective [41]. Clinical studies also report reduced post-operative discomfort, including diminished pain and faster symptom resolution, when Er:YAG laser irrigation is used instead of traditional or ultrasonic approaches [40,44]. Combining Er:YAG laser activation with photodynamic therapy (PDT) can yield bactericidal effects comparable or superior to standard NaOCl protocols, achieving up to 99.97% bacterial reduction and minimizing cytotoxic concerns [46]. Finally, Er:YAG laser treatment not only cleans canal walls effectively but also preserves or enhances dentinal surface properties conducive to cell attachment, making it a promising adjunct in regenerative endodontic procedures that require favorable stem cell adhesion and biocompatibility [39].

Other studies have reached similar conclusions regarding the effectiveness of laser-based treatments in dentistry. For instance, Otero (2024) demonstrated that laser-activated irrigation (LAI) using various wavelengths influences root canal smear-layer removal and dentin micromorphology in different ways [48]. Specifically, Er:YAG lasers partially cleaned the smear layer, while CO₂ and diode lasers at certain power settings either cleaned and opened dentinal tubules or melted and sealed them with recrystallized material [48]. In addition to endodontic applications, Er:YAG lasers have proven beneficial in conservative and restorative dentistry [48,49]. Fornaini (2013) showed that Er:YAG lasers can serve as an effective alternative to traditional turbine and micro-motor tools in adhesive procedures. When combined with orthophosphoric acid, Er:YAG laser treatment improved bond strength, reduced microleakage, diminished patient discomfort, and led to higher satisfaction levels [25]. Er:YAG lasers have also been investigated for periodontal applications [25]. Aoki et al. found that Er:YAG laser debridement of root surfaces and bone defects is both safe and effective, delivering results that are comparable or superior to those of conventional mechanical methods [49]. Moreover, their findings highlighted the Er:YAG laser’s utility as a preparatory device in periodontal flap surgery [30]. Similarly, Yaneva et al. showed that Er:YAG laser use in non-surgical periodontal therapy is clinically equivalent to hand instrumentation and can provide enhanced long-term stability [50]. Beyond periodontal treatment, Er:YAG lasers offer advantages in orthodontic care. Al-Jundi et al. concluded that Er:YAG laser application accelerates orthodontic tooth movement while significantly reducing post-treatment pain and discomfort [51]. In implant dentistry, Kriechbaumer et al. demonstrated that Er:YAG lasers enable precise, non-toxic, and highly effective removal of biofilm from metal implants, thereby presenting clear benefits over conventional disinfectants in infection control and implant retention procedures [52]. Further supporting its versatility, Taniguchi et al. revealed that Er:YAG laser-assisted bone regenerative therapy (Er-LBRT) without barrier membranes can achieve favorable ridge preservation (RP) and ridge augmentation (RA). This approach promoted sufficient bone regeneration for implant placement and ensured successful wound healing along with long-term peri-implant stability [53]. Stübinger similarly recognized the Er:YAG laser’s 2.94 μm wavelength—matching the water absorption peak—as an effective alternative to conventional bone ablation methods. Although modern short-pulsed Er:YAG systems address many earlier concerns (e.g., tissue damage, delayed healing, and extended osteotomy times), limitations such as depth control and safe beam guidance still hinder their routine use in oral and implant surgery [54]. Finally, Li et al. reported that Er:YAG lasers can deliver health benefits for peri-implantitis patients, notably by reducing probing depth (PD) and gingival recession (GR). However, they stressed the need for further research to confirm these promising results due to the inherent limitations of the included studies [55,56,57,58]. Collectively, these findings underscore the expanding role of Er:YAG lasers in dentistry—from endodontics to periodontics, orthodontics, and implantology—while also emphasizing the necessity of continued research and technological refinement to optimize clinical outcomes.

### 4.2. Limitations of the Evidence

Despite the overall positive outcomes reported, several limitations in the existing body of evidence on Er:YAG laser-assisted root canal disinfection preclude drawing definitive conclusions. First, heterogeneity in study designs and protocols—such as variations in laser settings, irrigation regimens, and outcome measurements—makes direct comparisons difficult and complicates attempts at meta-analysis. Second, while in vitro and ex vivo experiments offer mechanistic insights, their clinical applicability may be limited by small sample sizes and the absence of in vivo conditions such as tissue fluid or patient factors. Third, many included clinical studies feature modest participant numbers, short follow-up periods, or no standardized measures of long-term success (e.g., radiographic or histological healing). Fourth, the lack of uniform protocols for bacterial assessment—ranging from colony-forming units to molecular methods—introduces further variability in the reported efficacy. Finally, restricting the review to English-language and peer-reviewed publications, along with potential inconsistencies in blinding and randomization, raises the risk of selection and publication bias.

### 4.3. Limitations of the Review Process

Despite adhering to PRISMA guidelines and employing a thorough search strategy across multiple databases, the review process itself is subject to several limitations. First, restricting the included studies to those published in English may introduce language bias and potentially exclude relevant research in other languages. Second, the exclusion of grey literature—such as conference proceedings, theses, and unpublished studies—can lead to publication bias, as positive findings are more frequently published than negative or inconclusive results. Third, the substantial heterogeneity in study designs, laser protocols, and outcome measures among the included papers limited the feasibility of conducting a quantitative meta-analysis and necessitated a largely narrative synthesis of findings. Additionally, although dual screening and data extraction were performed to reduce selection bias, subjectivity in interpreting study methodologies and outcomes remains a concern. Finally, the absence of a uniform quality-rating framework, beyond the presented risk-of-bias checklist, further constrains the ability to make broad, definitive conclusions regarding the efficacy of Er:YAG laser-assisted root canal disinfection.

### 4.4. Implications for Practice, Policy, and Future Research

Er:YAG laser technology holds considerable promise for elevating clinical outcomes in root canal disinfection, yet its integration into mainstream practice requires further standardization and research. For clinicians, the review findings underscore the potential of Er:YAG lasers to facilitate deeper irrigant penetration, effectively remove smear layers, and minimize bacterial loads—even in complex anatomies—thereby enhancing both immediate disinfection and long-term prognosis. Policy-wise, professional bodies, and regulatory agencies could develop evidence-based guidelines on Er:YAG laser parameters (e.g., energy settings, pulse duration) and optimal adjunctive irrigants, ensuring safe and standardized application in various endodontic scenarios. Future research should prioritize larger-scale, multicenter clinical trials with extended follow-up intervals to capture long-term success and cost-effectiveness, while also exploring specialized areas such as pediatric endodontics or regenerative procedures. Additionally, harmonizing study methodologies—ranging from bacterial assessment techniques to operator training—would enable more robust comparisons across research and lay a more consistent foundation for the clinical deployment of Er:YAG laser-assisted irrigation.

This study adds to the existing body of knowledge by systematically reviewing and comparing the effectiveness of Er:YAG laser-assisted root canal disinfection against conventional and other laser-based methods. While previous studies have explored various laser applications in endodontics, there remains a lack of consensus regarding standardized protocols and optimal laser parameters for effective disinfection. A key novelty of this study is its focus on recent advancements in Er:YAG laser technology, specifically PIPS, and SWEEPS, which have demonstrated enhanced fluid dynamics and deeper irrigant penetration. Unlike diode and Nd:YAG lasers, which primarily rely on thermal effects, the Er:YAG laser’s ability to generate shockwaves without excessive heat reduces the risk of structural damage to dentin while achieving superior bacterial reduction. Additionally, this review highlights the potential for Er:YAG laser technology to enable the use of lower concentrations of sodium hypochlorite without compromising antimicrobial efficacy. This is particularly relevant in minimizing the cytotoxic effects associated with chemical irrigants. Furthermore, by identifying gaps in current research, this study sets the stage for future investigations into long-term clinical outcomes, cost-effectiveness, and potential applications of Er:YAG lasers in regenerative endodontic procedures and pediatric dentistry. Overall, this study advances the field by providing a comprehensive analysis of Er:YAG laser-assisted disinfection, emphasizing its unique advantages over other laser types and proposing recommendations for optimized clinical implementation.

## 5. Conclusions

Er:YAG laser-assisted irrigation outperforms conventional methods, providing better bacterial reduction, deeper irrigant penetration, and improved smear layer removal. PIPS and SWEEPS further enhance fluid dynamics and biofilm disruption, reducing reliance on high NaOCl concentrations. However, variability in settings and limited long-term data highlight the need for standardized protocols and larger studies. Er:YAG lasers offer superior disinfection with minimal thermal damage, making them valuable in endodontics and periodontics for minimally invasive bacterial decontamination. Their use in pediatric and regenerative endodontics also supports tissue preservation and effective microbial control. Challenges include high costs, specialized training requirements, and parameter inconsistencies, necessitating further research. Despite this, Er:YAG lasers show strong potential for improving root canal disinfection and long-term treatment outcomes, positioning them as a viable alternative to conventional methods.

## Figures and Tables

**Table 1:** Search syntax used in the study.

Source	Search Term	No.
PubMed/MEDLINE	(“root canal disinfection”[Title/Abstract] OR “root canal therapy”[MeSH Terms] OR “endodontic treatment”[Title/Abstract] OR “endodontics”[MeSH Terms] OR “root canal irrigation”[MeSH Terms] OR “endodontic irrigation”[Title/Abstract] OR “antibacterial endodontic therapy”[Title/Abstract]) AND (“Er:YAG laser”[Title/Abstract] OR “erbium-doped yttrium aluminum garnet laser”[Title/Abstract] OR “Erbium lasers”[MeSH Terms] OR “Erbium:YAG laser”[Title/Abstract] OR “Er:YAG laser therapy”[Title/Abstract] OR “Er:YAG laser irradiation”[Title/Abstract] OR “laser-activated irrigation”[Title/Abstract] OR “photoacoustic streaming”[Title/Abstract] OR “PIPS”[Title/Abstract] OR “SWEEPS”[Title/Abstract]) AND (“disinfection”[Title/Abstract] OR “antibacterial”[Title/Abstract] OR “sterilization”[MeSH Terms] OR “antimicrobial”[Title/Abstract] OR “biofilm removal”[Title/Abstract] OR “bacterial reduction”[Title/Abstract] OR “irrigation activation”[Title/Abstract] OR “root canal sterilization”[Title/Abstract] OR “smear layer removal”[Title/Abstract])	28
Embase	((’root canal disinfection’:ti,ab OR ’root canal therapy’/exp OR ’endodontic treatment’:ti,ab OR ’endodontics’/exp OR ’root canal irrigation’/exp OR ’endodontic irrigation’:ti,ab OR ’antibacterial endodontic therapy’:ti,ab OR ’root canal sterilization’:ti,ab) AND (’Er:YAG laser’:ti,ab OR ’erbium-doped yttrium aluminum garnet laser’:ti,ab OR ’Erbium lasers’/exp OR ’Erbium:YAG laser’:ti,ab OR ’Er:YAG laser therapy’:ti,ab OR ’Er:YAG laser irradiation’:ti,ab OR ’laser-activated irrigation’:ti,ab OR ’photoacoustic streaming’:ti,ab OR ’photon-induced photoacoustic streaming’:ti,ab OR ’shock wave-enhanced emission photoacoustic streaming’:ti,ab OR ’PIPS’:ti,ab OR ’SWEEPS’:ti,ab) AND (’disinfection’:ti,ab OR ’antibacterial’:ti,ab OR ’sterilization’/exp OR ’antimicrobial’:ti,ab OR ’biofilm removal’:ti,ab OR ’bacterial reduction’:ti,ab OR ’irrigation activation’:ti,ab OR ’root canal sterilization’:ti,ab OR ’smear layer removal’:ti,ab OR ’bacterial decontamination’:ti,ab))	24
Scopus	(TITLE-ABS(“root canal disinfection”) OR TITLE-ABS(“root canal therapy”) OR TITLE-ABS(“endodontics”) OR TITLE-ABS(“endodontic treatment”) OR TITLE-ABS(“root canal irrigation”) OR TITLE-ABS(“endodontic irrigation”) OR TITLE-ABS(“antibacterial endodontic therapy”) OR TITLE-ABS(“root canal sterilization”)) AND (TITLE-ABS(“Er:YAG laser”) OR TITLE-ABS(“erbium-doped yttrium aluminum garnet laser”) OR TITLE-ABS(“Erbium:YAG laser”) OR TITLE-ABS(“Erbium lasers”) OR TITLE-ABS(“Er:YAG laser therapy”) OR TITLE-ABS(“Er:YAG laser irradiation”) OR TITLE-ABS(“laser-activated irrigation”) OR TITLE-ABS(“photoacoustic streaming”) OR TITLE-ABS(“photon-induced photoacoustic streaming”) OR TITLE-ABS(“shock wave-enhanced emission photoacoustic streaming”) OR TITLE-ABS(“PIPS”) OR TITLE-ABS(“SWEEPS”)) AND (TITLE-ABS(“disinfection”) OR TITLE-ABS(“antibacterial”) OR TITLE-ABS(“sterilization”) OR TITLE-ABS(“antimicrobial”) OR TITLE-ABS(“biofilm removal”) OR TITLE-ABS(“bacterial reduction”) OR TITLE-ABS(“irrigation activation”) OR TITLE-ABS(“smear layer removal”) OR TITLE-ABS(“bacterial decontamination”))	28
Cochrane	((mh “Root Canal Therapy” OR mh “Endodontics” OR “root canal disinfection” OR “root canal therapy” OR “endodontic treatment” OR “root canal irrigation” OR “endodontic irrigation” OR “antibacterial endodontic therapy” OR “root canal sterilization”) AND (mh “Er:YAG laser” OR “Er:YAG laser” OR “erbium-doped yttrium aluminum garnet laser” OR “Erbium:YAG laser” OR “Erbium lasers” OR “Er:YAG laser therapy” OR “Er:YAG laser irradiation” OR “laser-activated irrigation” OR “photoacoustic streaming” OR “photon-induced photoacoustic streaming” OR “shock wave-enhanced emission photoacoustic streaming” OR “PIPS” OR “SWEEPS”) AND (mh “Sterilization” OR “disinfection” OR “antibacterial” OR “antimicrobial” OR “biofilm removal” OR “bacterial reduction” OR “irrigation activation” OR “smear layer removal” OR “bacterial decontamination”))	7

**Table 3:** Summary of the Studies.

Author and Year	Country	Study Type	Study Overview
Afkhami et al. 2021 [34]	Iran	In vitro	Comparative study on silver nanoparticle irrigation activation methods
Bao et al. 2024 [35]	China	In vitro	In vitro study comparing Er:YAG laser irrigation techniques
Deleu et al. 2015 [36]	Belgium	In vitro	In vitro study on laser-based irrigation methods
Ozbay et al. 2018 [37]	India	In vitro	In vitro study on smear layer removal with laser activation
Lei et al. 2022 [38]	China	In vitro	In vitro study using SWEEPS laser modality in root canal irrigation
Liu et al. 2023 [39]	Japan	In vitro	Comparative study on root canal irrigation beyond fractured instruments
Mandras et al. 2020 [40]	Italy	RCT	Randomized clinical trial on PIPS irrigation and post-operative effects
Murugesh Yavagal et al. 2021 [41]	India	Ex vivo	Ex vivo study on laser disinfection in pediatric root canals
Neelakantan et al. 2015 [42]	India	In vitro	In vitro study on antibiofilm activity of irrigation protocols
Rahmati et al. 2022 [43]	Iran	In vitro	SEM study on stem cell adhesion with Er:YAG laser and irrigants
Shan et al. 2022 [44]	China	In vitro	Comparison of Er:YAG laser and ultrasonic irrigation techniques
Todea et al. 2018 [45]	Romania	In vitro	SEM evaluation of Er:YAG laser on root canal morphology
Yang et al. 2024 [46]	China	In vitro	In vitro study on Er:YAG laser-activated PDT for root canal disinfection
Zhao et al. 2024 [47]	China	RCT	Randomized clinical trial comparing PIPS and conventional irrigation for apical periodontitis

RCT—Randomised clinical trial.

**Table 4:** Main outcomes and study groups.

Author and Year	Study Groups	Main Outcomes
Afkhami et al. 2021 [34]	AN Group: AgNPs alone. AN/ICG/DL Group: AgNPs + indocyanine green (ICG) + diode laser activation. AN/PIPS Group: AgNPs + photon-induced photoacoustic streaming (Er:YAG laser). AN/MDA Group: AgNPs + manual dynamic activation (gutta-percha). AN/PUI Group: AgNPs + passive ultrasonic irrigation. Positive Control: 5% NaOCl. Negative Control: No treatment.	The study investigated the effectiveness of Er:YAG laser technology, specifically through PIPS, in enhancing root canal disinfection. The findings revealed that PIPS significantly improved the efficacy of AgNP irrigants, achieving a bacterial reduction rate of 91.03% against *Enterococcus faecalis*. This technique generated shockwaves and mechanical energy, allowing better penetration and activation of the irrigants without excessive heat generation or the need for extensive canal preparation. PIPS outperformed other activation methods, including manual dynamic activation, passive ultrasonic irrigation, and photodynamic therapy, making it a promising adjunct for improving the disinfection of complex root canal systems.
Bao et al. 2024 [35]	CNI: Irrigation with a syringe and side-vented needle using 3% NaOCl. PUI: Agitation of NaOCl using an ultrasonic device with a K25 endodontic tip. EDDY: Sonic activation of NaOCl with a polymer EDDY tip powered by an air scaler. PIPS: Er:YAG laser-activated irrigation using a specialized PIPS fiber tip. SWEEPS: Er:YAG laser-activated irrigation using a SWEEPS fiber tip. Control Group: No irrigation or biofilm removal intervention was performed.	Er:YAG LAI (PIPS, SWEEPS), were more effective in disinfecting root canals compared to traditional methods such as CNI, PUI, and EDDY. SWEEPS exhibited the highest efficacy in removing biofilms from challenging areas like apical artificial grooves and dentinal tubules, followed closely by PIPS and EDDY. The enhanced performance of these laser methods was attributed to their ability to generate dynamic fluid motion and penetrate hard-to-reach regions.
Deleu et al. 2015 [36]	CI: Syringe irrigation with a 27-gauge needle. MDI: Gutta-percha cone with push-pull strokes. PUI: Oscillating non-cutting ultrasonic file. Er:YAG Laser: Plain fiber tip inside the canal. Er:YAG Laser with PIPS Tip: Conical radial-firing fiber tip at the canal entrance. 980-nm Diode Laser: Plain fiber tip moving along the canal.	The findings demonstrated that the Er:YAG laser with a plain fiber tip inside the canal was significantly more effective in removing debris from simulated root canal irregularities compared to conventional irrigation, manual-dynamic irrigation, and a 980-nm diode laser. The efficacy of the Er:YAG laser was attributed to cavitation effects, including the rapid formation and implosion of vapor bubbles at the fiber tip, which generated shock waves and fluid movement that improved debris removal from areas not reachable by traditional methods.
Ozbay et al. 2018 [37]	Control Group: No irrigation during preparation. Experimental Groups: Group I: Largal Ultra as the final irrigant: IA: Without laser activation. IB: With Nd:YAG laser activation. IC: With Er:YAG laser activation. Group II: Biopure MTAD as the final irrigant: IIA: Without laser activation. IIB: With Nd:YAG laser activation. IIC: With Er:YAG laser activation.	Laser-activated irrigation improved smear layer removal and sealer penetration into dentinal tubules, with Er:YAG laser showing superior performance over Nd:YAG. The use of Biopure MTAD as an irrigant was more effective than Largal Ultra, and laser activation further amplified the benefits, particularly with the Er:YAG laser due to its ability to create photoacoustic shockwaves that improve irrigation efficiency. Despite these advances, neither laser completely removed the smear layer or achieved full sealer penetration in the apical thirds of curved root canals, highlighting the potential for further optimization in clinical practice.
Lei et al. 2022 [38]	NS + CI: Normal saline with conventional needle irrigation. NS + SWEEPS: Normal saline with SWEEPS. 0.5% NaOCl + SWEEPS: 0.5% sodium hypochlorite with SWEEPS. 1% NaOCl + SWEEPS: 1% sodium hypochlorite with SWEEPS. 2% NaOCl + SWEEPS: 2% sodium hypochlorite with SWEEPS. 5.25% NaOCl + SWEEPS: 5.25% sodium hypochlorite with SWEEPS	Root canal disinfection with Er:YAG laser technology, particularly using the SWEEPS modality, demonstrated its effectiveness in improving disinfection efficacy. SWEEPS significantly enhanced the antimicrobial activity of low-concentration NaOCl solutions by generating strong photoacoustic currents and mechanical agitation, facilitating deeper irrigant penetration and increased interaction with bacteria. The results indicated that SWEEPS-assisted irrigation could achieve comparable bacterial reduction across various NaOCl concentrations, suggesting its potential to maintain effective disinfection at safer, lower NaOCl concentrations. This synergistic effect reduces the risk of tissue damage and improves the safety profile of root canal treatments.
Liu et al. 2023 [39]	LAI-PIPS: Used a 2.94 μm Er:YAG laser unit at 20 mJ/15 Hz with the tip positioned 2 mm from the canal orifice. LAI: Used an Er:YAG laser unit at 30 mJ/20 Hz with the tip inserted 8 mm from the canal orifice. UAI: Used an ultrasonic file at 30 kHz/3.6 W with the tip inserted 8 mm from the canal orifice. SI: Used a standard needle for irrigant delivery.	The study on enhancing root canal disinfection with Er:YAG laser technology concluded that both LAI and LAI-PIPS demonstrated superior cleaning efficacy in removing debris and smear layers in the apical area, even beyond fractured instruments, compared to UAI and conventional syringe irrigation. LAI-PIPS, which operates at lower power with a shorter pulse width, produced a “shockwave-like” effect, resulting in enhanced debris removal and cleaning of narrow apical regions. Both LAI and LAI-PIPS also exhibited significantly higher vapor bubble velocities and counts, indicating more dynamic irrigant activity, while LAI-PIPS showed the best performance overall. These findings highlight the potential of Er:YAG laser technology in improving root canal disinfection, particularly in challenging cases with fractured instruments
Mandras et al. 2020 [40]	Group A (Control Group): Received traditional endodontic manual irrigation using 5% NaOCl and 10% EDTA with a 30G side-vented steel needle. Group B (Experimental Group): Received PIPS irrigation with an Er:YAG laser, using the same irrigants (5% NaOCl and 10% EDTA) activated by laser pulses in a standardized protocol.	The study demonstrated that the use of the Er:YAG laser with PIPS significantly enhanced bacterial load reduction in root canal disinfection compared to traditional irrigation methods. PIPS showed superior antibacterial efficacy, particularly against facultative anaerobes and Gram-negative obligate anaerobes, without increasing the risk of irrigant extrusion or debris beyond the apex. Post-operative QoL measures indicated less discomfort, including reduced pain, difficulty eating, and daily functioning during the initial recovery phase. While PIPS provided effective disinfection with favourable patient outcomes, the differences in efficacy compared to traditional methods were not statistically significant for all bacterial strains, highlighting its potential as a complementary disinfection strategy in endodontic treatments.
Murugesh Yavagal et al. 2021 [41]	Group 1 (CI): Root canals were irrigated using 2.5% NaOCl with a syringe, placed in the middle third of the canal, without activation, and with a resting time of 30 s between cycles. Group 2 (LAI): Root canals were irrigated using an Er:YAG laser (wavelength 2940 nm) with NaOCl. The laser was set to 50 μs pulse duration, 15 Hz pulse rate, and 20 mJ energy, with the laser tip placed in the coronal pulp chamber. Irrigation involved 30-s activation intervals for three cycles.	Significant efficacy of Er:YAG laser technology, specifically PIPS, in enhancing root canal disinfection in primary teeth. It demonstrated complete elimination of *Enterococcus faecalis* in canals treated with laser-activated NaOCl irrigation, outperforming conventional syringe irrigation with NaOCl. The laser’s mechanism, which generates microcavitation and shockwaves, promotes thorough 3D streaming of the irrigant, reaching areas inaccessible to conventional methods. This approach was shown to overcome challenges posed by the complex anatomy of primary teeth, providing effective, minimally invasive disinfection while avoiding thermal damage to dentinal walls. PIPS is a promising innovation for pediatric endodontics, particularly for achieving complete bacterial eradication in challenging root canal systems.
Neelakantan et al. 2015 [42]	Irrigation Protocols: NaOCl + Etidronic Acid: 1:1 mixture of 6% NaOCl and 18% etidronic acid. NaOCl-EDTA: 3% NaOCl followed by 17% EDTA. NaOCl-EDTA-NaOCl: 3% NaOCl, 17% EDTA, and a final flush of 3% NaOCl. Control Group: Saline irrigation. Activation Methods (applied within each irrigation group): A. No Activation B. Ultrasonic Activation C. Diode Laser ActivationEr:YAG Laser Activation	The study highlights that Er:YAG laser activation significantly enhances the disinfection of root canals by facilitating the destruction of mature *Enterococcus faecalis* biofilms and improving bacterial reduction in dentinal tubules when compared to conventional syringe irrigation and ultrasonic activation. Among various irrigation protocols, the combination of NaOCl with etidronic acid or NaOCl-EDTA-NaOCl showed the highest efficacy in bacterial elimination, particularly when activated by Er:YAG laser or diode laser. The photoacoustic streaming effect of the Er:YAG laser was particularly effective in increasing the penetration and reaction kinetics of NaOCl, leading to better biofilm disruption and dentinal tubule disinfection, outperforming other activation methods.
Rahmati et al. 2022 [43]	Control Group: No irrigation or laser treatment was applied. EDTA Group: Irrigated with 17% EDTA for 1 min. MTAD Group: Irrigated with MTAD for 5 min. QMix Group: Irrigated with QMix for 5 min. Laser Group: Treated with Er:YAG laser irradiation at 2.94 µm wavelength, 25 mJ energy per pulse, 15 Hz frequency, for 20 s.	The Er:YAG laser, EDTA, and QMix promoted SCAP adhesion similarly, while MTAD significantly inhibited adhesion. The Er:YAG laser emerged as a promising technology due to its ability to expose collagen fibers and create porosities on the dentin surface, enhancing cell attachment. EDTA effectively eliminated the smear layer and supported SCAP adhesion through the exposure of growth factors, while QMix demonstrated a favorable combination of smear layer removal, antimicrobial activity, and biocompatibility. Conversely, despite its antimicrobial properties, MTAD was not recommended for regenerative procedures as it impaired cell attachment, highlighting the importance of selecting agents that support stem cell adhesion in tissue engineering.
Shan et al. 2022 [44]	Access Types: CIA: Traditional larger access to the root canal. MIA: Smaller, computer-guided conservative access. Disinfection Methods: CI: Manual irrigation using NaOCl and EDTA solutions. PUI: Ultrasonic-assisted activation of irrigants. Er:YAG (LAI): Laser-assisted activation of irrigants.	The study compared the disinfection efficacy of Er:YAG laser, ultrasonic, and conventional irrigation methods in root canals accessed through both conventionally and minimally invasive approaches. The Er:YAG laser demonstrated superior bacterial removal, especially in dentinal tubules, due to its enhanced irrigant penetration and photothermal effects. Both Er:YAG laser and ultrasonic methods were significantly more effective than conventional irrigation, and their efficacy was similar irrespective of the invasiveness of the access. The Er:YAG laser showed practical advantages, such as ease of operation and reduced risk of instrument contact with canal walls, making it particularly suitable for minimally invasive treatments. These findings highlight the potential of Er:YAG laser technology to optimize root canal disinfection in contemporary minimally invasive endodontics.
Todea et al. 2018 [45]	Control Group: Two teeth underwent conventional root canal treatment without laser irradiation. Group 2: Twelve teeth were irradiated with the Er:YAG laser using PIPS at 10 mJ, 10 Hz, and VSP mode for 10 s, repeated three times per tooth under 2.5% NaOCl irrigation. Group 3: Twelve teeth were irradiated with the Er:YAG laser using PIPS at 20 mJ, 15 Hz, and SSP mode for 10 s, repeated four times per tooth, also under 2.5% NaOCl irrigation	The studies highlight that Er:YAG laser technology significantly enhances root canal disinfection by effectively removing debris and smear layers from dentinal tubules, surpassing the results of conventional methods. Utilizing PIPS tips, the Er:YAG laser demonstrated superior cleaning efficacy across all root canal sections—cervical, middle, and apical—without altering the root canal morphology. This advanced technique improves the permeability of dentinal tissue, opens dentinal tubules, and reaches collateral canals, which are typically inaccessible with standard irrigation methods. Additionally, the combination of Er:YAG laser with sodium hypochlorite irrigation amplified bactericidal effects, addressing even deeply seated infections like *Enterococcus faecalis*. When operated at subablative parameters, the laser ensures minimal thermal or structural damage while providing uniform and enhanced disinfection, making it a promising addition to modern endodontic protocols.
Yang et al. 2024 [46]	Control Group: No treatment was applied. NaOCl Group: Root canals were irrigated with 2.5% NaOCl for 2 min. NaOCl + Er:YAG Group: NaOCl irrigation followed by Er:YAG laser activation (40 mJ, 0.4 W, 10 Hz, for 1 min). PDT Group: Root canals were treated with methylene blue (50 µmol/L) followed by 660 nm laser irradiation for 1 min. PDT + Er:YAG Group: Methylene blue was activated with an Er:YAG laser (same parameters as above) and subsequently irradiated with a 660 nm laser for 1 min.	The study demonstrates that the use of Er:YAG laser technology in combination with PDT significantly enhances root canal disinfection, particularly against Enterococcus faecalis. While PDT alone achieved an 87.22% reduction in bacterial colonies, its efficacy was notably improved when paired with Er:YAG laser activation, achieving a 99.97% reduction. This approach showed bactericidal effects comparable to NaOCl activated by Er:YAG laser, offering a potential alternative to traditional methods. The findings underscore the Er:YAG laser’s ability to improve photosensitizer penetration in dentin tubules, enhancing disinfection efficacy while potentially mitigating the cytotoxic risks associated with NaOCl.
Zhao et al. 2024 [47]	CNI Group: Teeth underwent root canal irrigation using 1% NaOCl activated by CNI. The irrigation was performed by inserting a 30-gauge side needle tip to 1 mm short of the working length for 30 s with 3 mL of NaOCl. PIPS Group: Teeth underwent root canal irrigation using 1% NaOCl activated by PIPS with an Er:YAG laser. The laser tip was positioned in the pulp chamber and activated for 30 s with 3 mL of NaOCl, utilizing a 2940 nm laser with specific settings (0.3 W, 15 Hz, 20 mJ per pulse).	PIPS with an Er:YAG laser significantly enhanced root canal disinfection compared to CNI when both used 1% NaOCl. PIPS showed superior antibacterial efficacy, as indicated by lower ATP values post-irrigation, and led to improved clinical outcomes, including reduced percussion tenderness and resolution of fistulas after seven days. The enhanced disinfection with PIPS was attributed to its ability to generate strong shockwaves and effectively remove biofilms from the apical region. While both methods reduced symptoms, PIPS outperformed CNI in bacterial elimination and potentially in clinical healing, though larger studies are suggested for more definitive conclusions.

Silver nanoparticles (AgNPs); Indocyanine green (ICG); Diode laser activation (DI); Photon-induced photoacoustic streaming (PIPS); Manual dynamic activation (MDA); Sodium hypochlorite (NaOCl); Conventional Needle Irrigation (CNI); Passive Ultrasonic Irrigation (PUI); Sonic-Powered Irrigation with a polymer tip (EDDY); Shock Wave-Enhanced Emission Photoacoustic Streaming (SWEEPS); Manual-Dynamic Irrigation (MDI); Er:YAG Laser with plain fiber tip inside the canal (Er-flat); Er:YAG Laser with conical radial-firing fiber tip (Er-PIPS); Normal saline (NS); Laser-Activated Irrigation with PIPS (NLAI-PIPS); Laser-Activated Irrigation (LAI); Ultrasonic-Activated Irrigation (UAI); Syringe Irrigation (SI); Sequential irrigants (NaOCl-EDTA-NaOCl); Ethylenediaminetetraacetic acid (EDTA); Mixture of tetracycline isomer, acid, detergent (MTAD); CHX + EDTA + surfactant (QMix); Conventionally Invasive Access (CIA); Minimally Invasive Access (MIA); Variable Square Pulse mode (VSP); Super Short Pulse mode (SSP); Photodynamic Therapy (PDT); Quality of life (QoL).

**Table 5:** Summary of Er:YAG laser parameters from each study.

Author/Year	Operating Mode	Wavelength (nm)	Power Output (mW)	Irradiation Time (s)
Afkhami et al. 2021 [34]	Pulsed mode	2940	300	30
Bao et al. 2024 [35]	PIPS SWEEPS	2940	PIPS: 300 mW SWEEPS: 600 mW	30
Deleu et al. 2015 [36]	-	2940	-	5 s per activation, repeated 4 times.
Ozbay et al. 2018 [37]	MSP	2940	1000	10
Lei et al. 2022 [38]	Auto-SWEEPS mode with synchronized double pulses	2940	300	30
Liu et al. 2023 [39]	LAI-PIPS and LAI.	2940	20 mJ at 15 Hz for LAI-PIPS and 30 mJ at 20 Hz for LAI.	5
Mandras et al. 2020 [40]	SSP		300	30
Murugesh Yavagal et al. 2021 [41]	Pulsed	2940	300	30
Neelakantan et al. 2015 [42]	Pulsed	2940		30
Rahmati et al. 2022 [43]		2940		20
Shan et al. 2022 [44]	SSP	2940	300	30
Todea et al. 2018 [45]	Group 2: VSP mode Group 3: SSP mode	2940	Group 2: 10 mJ, 10 Hz; Group 3: 20 mJ, 15 Hz.	10
Yang et al. 2024 [46]	Root canal shaking mode	2940	400	60
Zhao et al. 2024 [47]	PIPS	2940	300	30

PIPS: Photon-Induced Photoacoustic Streaming; SWEEPS: Shock Wave-Enhanced Emission Photoacoustic Streaming; MSP: Micro Short Pulse; LAI-PIPS: Laser-Activated Irrigation with Photon-Induced Photoacoustic Streaming; LAI: Laser-Activated Irrigation; VSP: Variable Square Pulse; SSP: Super Short Pulse.

## Data Availability

No new data were created or analyzed in this study.
